# Radiological prognostic factors of chronic subdural hematoma recurrence: a systematic review and meta-analysis

**DOI:** 10.1007/s00234-020-02558-x

**Published:** 2020-10-22

**Authors:** Ishita P. Miah, Yeliz Tank, Frits R. Rosendaal, Wilco C. Peul, Ruben Dammers, Hester F. Lingsma, Heleen M. den Hertog, Korné Jellema, Niels A. van der Gaag, on behalf of the Dutch Chronic Subdural Hematoma Research Group

**Affiliations:** 1 Department of Neurology and Neurosurgery, Leiden University Medical Center (LUMC), Albinusdreef 2, 2333 ZA Leiden, The Netherlands; 2 Department of Radiology, Leiden University Medical Center (LUMC), Albinusdreef 2, 2333 ZA Leiden, The Netherlands; 3 Department of Clinical Epidemiology, Leiden University Medical Center (LUMC), Albinusdreef 2, 2333 ZA Leiden, The Netherlands; 4 Department of Neurology and Neurosurgery, Haaglanden Medical Center (HMC), Lijnbaan 32, 2512 VA The Hague, The Netherlands; 5 Department of Neurosurgery, Erasmus Medical Center (EMC), Erasmus MC Stroke Center, Dr. Molewaterplein 40, 3015 GD Rotterdam, The Netherlands; 6 Department of Public Health and Medical Decision Making, Erasmus Medical Center (EMC), Dr. Molewaterplein 40, 3015 GD Rotterdam, The Netherlands; 7 Department of Neurology, Isala Hospital Zwolle, Dokter van Heesweg 2, 8025 AB Zwolle, The Netherlands; 8 Department of Neurosurgery, Haga Teaching Hospital, Els Borst-Eilersplein 275, 2545 AA The Hague, The Netherlands

**Keywords:** Chronic subdural hematoma, CSDH, Recurrence, Predictors, CT

## Abstract

**Purpose:**

Chronic subdural hematoma (CSDH) is associated with high recurrence rates. Radiographic prognostic factors may identify patients who are prone for recurrence and who might benefit further optimization of therapy. In this meta-analysis, we systematically evaluated pre-operative radiological prognostic factors of recurrence after surgery.

**Methods:**

Electronic databases were searched until September 2020 for relevant publications. Studies reporting on CSDH recurrence in symptomatic CSDH patients with only surgical treatment were included. Random or fixed effects meta-analysis was used depending on statistical heterogeneity.

**Results:**

Twenty-two studies were identified with a total of 5566 patients (mean age 69 years) with recurrence occurring in 801 patients (14.4%). Hyperdense components (hyperdense homogeneous and mixed density) were the strongest prognostic factor of recurrence (pooled RR 2.83, 95% CI 1.69–4.73). Laminar and separated architecture types also revealed higher recurrence rates (RR 1.37, 95% CI 1.04–1.80 and RR 1.76 95% CI 1.38–2.16, respectively). Hematoma thickness and midline shift above predefined cut-off values (10 mm and 20 mm) were associated with an increased recurrence rate (RR 1.79, 95% CI 1.45–2.21 and RR 1.38, 95% CI 1.11–1.73, respectively). Bilateral CSDH was also associated with an increased recurrence risk (RR 1.34, 95% CI 0.98–1.84).

**Limitations:**

Limitations were no adjustments for confounders and variable data heterogeneity. Clinical factors could also be predictive of recurrence but are beyond the scope of this study.

**Conclusions:**

Hyperdense hematoma components were the strongest prognostic factor of recurrence after surgery. Awareness of these findings allows for individual risk assessment and might prompt clinicians to tailor treatment measures.

## Introduction

Chronic subdural hematoma (CSDH) is a frequently encountered neurosurgical disorder of the elderly with a rising incidence [1, 2]. Historically, CSDH was considered as a progressive and recurrent hemorrhage due to rupture of cortical bridging veins initiated by trauma [3]. Recently however, it has been suggested that a more complex pathway of inflammation, angiogenesis, recurrent micro-hemorrhages, and local coagulopathy in the subdural space is involved [4–8]. This inflammatory response is presumed to play a key role in hematoma formation, re-bleeding, and maintenance.

The diagnosis is based on clinical symptoms and radiological investigation, mostly non-contrast CT scan. Surgery through burr hole drainage or twist drill craniostomy (BHC, TDC) is the mainstay of treatment worldwide [9, 10]. Alternative strategies include watchful observation or high-dose glucocorticoids administration depending on symptom severity and local protocols [11–14]. Ultimately, the aim of all therapeutic modalities is adequate symptom relieve by effective hematoma resolution.

Recurrence of CSDH after surgery occurs frequently with reported rates that vary between 2.5 and 33% [15–17]. Postoperative closed drainage as interventional measure is effective in reducing recurrence risk by roughly 50% [1, 10, 17]. Recurrent CSDH poses a formidable challenge in the treatment of symptomatic patients [18]. Recurring symptoms and additional treatment increase patient burden, prolong hospital admissions leading to higher costs, and contribute to a potential poor outcome [19, 20]. Therefore, the identification of factors associated with recurrence is important for individual risk assessment, treatment decisions, and possibly optimization of pre- and postoperative management. An individualized approach could entail adjusting the timing of surgery and anti-thrombotic therapy resumption or even exploring alternative treatment strategies depending on local protocols.

Many radiological parameters of CSDH have been reported to be associated with the recurrence risk, including uni- or bilateral hematoma, preoperative hematoma thickness and midline shift, hematoma density and internal architecture, cerebral atrophy, and hematoma volume [21–34]. However, studies have shown conflicting results and large discrepancies in recurrence rates due to heterogeneity in treatment, radiological measurement techniques, and variation in hematoma classifications for hematoma density or architecture.

In this systematic review and meta-analysis, we aimed to identify radiological prognostic factors of CSDH recurrence in surgically treated symptomatic CSDH patients.

## Materials and methods

Before conducting this systematic review, we developed a detailed protocol including objectives and a strategy for collecting and analyzing data. The manuscript was prepared in accordance with the Preferred Reporting Items for Systematic Review and Meta-analysis Protocols (PRISMA) guidelines.

### Search strategy and selection criteria

Literature on symptomatic CSDH patients and radiological findings published until September 2020 were reviewed using PubMed, EMBASE, Web of Science, and Cochrane library. Potential studies were searched using the following keywords and MeSH terms (including abbreviations, variations due to plurality and spelling): “chronic subdural hematoma,” “imaging,” “radiological,” “predictor,” “computed tomography,” and “magnetic resonance imaging.” The search was supplemented by hand searching the reference list of each included article and review article. Our primary outcome was CSDH recurrence. Inclusion criteria for study selection were the following: (1) symptomatic CSDH patients, (2) only surgical therapy by burr hole or twist-drill craniostomy with subdural drainage, (3) pre-defined (and retrievable) definition of CSDH recurrence, (4) follow-up period of ≥ 3 months, (5) clinical studies with > 10 subjects, and (6) evaluation of at least one of the following radiological parameters: uni- versus bilateral hematoma, hematoma thickness, midline shift, hematoma density and architecture on CT, hematoma volume, MRI appearance (T1, T2, diffusion-weighted imaging, DWI). Studies performed in animals, case reports or reported in other than English language were excluded.

### Data extraction

**Fig. 1:**
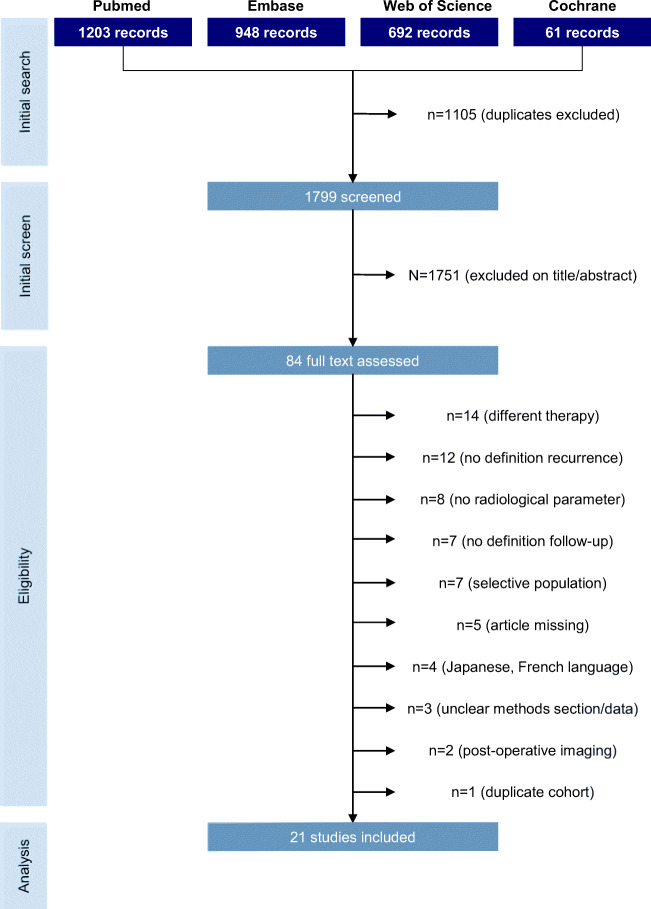
Hematoma architecture types: **a** homogeneous; **b** laminar; **c** separated; **d** trabecular type

Data from the included studies were extracted by one neurologist (IPM) and one radiologist (YT) using a standardized data extraction form. Disagreements were resolved by consensus. The following data were collected: (1) study characteristics (country, study design, year of publication, number of participants, definition of CSDH (diagnostic criteria) and CSDH recurrence, type of surgery, follow-up period, radiological parameters evaluated), (2) patient characteristics (mean age, sex, trauma, use of oral anticoagulation, or platelet aggregation inhibitors), and (3) imaging findings of radiological parameters: uni- versus bilateral hematoma, hematoma thickness (frequencies below or above prespecified cutoff value in mm), midline shift (present/absent or frequencies below or above prespecified cut-off value in mm), hematoma density classification and hematoma architecture types, volume (in mm^3^, frequencies above or below prespecified cut off value), and MRI appearance (hypo-, iso- or hyperintensity on T1, T2, and DW-imaging). Hematoma density was categorized as (1) homogeneous hypodense, (2) homogeneous isodense, (3) homogeneous hyperdense, and (4) mixed density. Hematoma architecture was reported using the four classification as described by Nakaguchi [26] (Table 1, Fig. 1): (a) homogeneous architecture, (b) laminar architecture, (c) separated architecture, and (d) trabecular architecture. Due to heterogeneity and lack of standardization in reporting on hematoma density and architecture, we added two simplified categories to summarize density and architecture findings: (i) a (total) homogeneous group containing all patients with a homogeneous hypodense, homogeneous isodense, and homogeneous hyperdense hematoma; (ii) (total) mixed density group, containing all mixed density hematoma and the following architecture types with mixed density: laminar, separated, grading, and trabecular hematoma.

**Table 1:** Hematoma classification by architecture type

Architecture types	Description
Homogeneous	Hematoma with complete homogeneous density, including homogeneous hypo-, iso-, and hyperdense hematoma
Laminar	Hematoma with thin high-density layer along the inner membrane (against the surface of the cortex)
Separated	Hematoma with two components of different densities with a clear boundary between them, resulting in a lower density component above a higher density component. If this boundary was mingled at the border, this was called a gradation type
Trabecular	Hematoma with inhomogeneous components and a high density septum running between the inner and outer hematoma membrane

### Quality of reporting in included studies

We assessed risk of bias and quality of reporting of all included studies based on the Newcastle–Ottawa Quality Assessment Scale (NOS) checklist, used to build a quality score between 0 and a maximum of 9 stars [35]. When there was risk of selection bias in patient inclusion (i.e., exclusion of patients with head trauma, anticoagulant or platelet aggregation inhibitor use, bilateral CSDH, or absence of follow-up CT), one star was subtracted in the selection-section (max. 4 stars). Stars were assigned in the comparability section if adjustments took place for confounders (max. 2 stars). If there was no statement regarding the number of patients who were actually evaluated at the predefined follow up moment (3, 6, or 12 months), one star was also subtracted in the outcome-section (max. 3 stars). Studies were rated with good quality if they had 3 or 4 stars in the selection domain and 1 or 2 stars in the comparability domain and 2 or 3 stars in the outcome/exposure domain. Studies were of fair quality when they scored 2 stars in the selection domain and 1 or 2 stars in the comparability domain and 2 or 3 stars in the outcome/exposure domain. Studies were also classified as fair quality when they had maximum stars in the selection and outcome domain, with no stars in the comparability section. Finally, studies were classified as poor quality when they scored 0 or 1 star in the selection domain or 0 stars in comparability domain or 0 or 1 star in outcome/exposure domain.

### Statistical analysis

Analyses were performed using SPSS (version 25.0, IBM Corp) and Review Manager (RevMan, version 5.3. Copenhagen: The Nordic Cochrane Center, The Cochrane Collaboration, 2014). Continuous and categorical variables were summarized with means and counts and percentages respectively. To evaluate recurrence risk, we calculated risk ratios (RR) with 95% confidence intervals for the following comparisons: (1) unilateral versus bilateral hematoma, (2) hematoma thickness below versus above prespecified cutoff values (15, 20, and 25 mm), (3) presence versus absence of midline shift, (4) midline shift below versus above prespecified cut off values (5, 10, and 15 mm), (5) mixed density (total) versus homogenous density (total) hematoma, (6) homogeneous hyper- and mixed density versus homogeneous iso- and hypodensity hematoma, (7) architecture types (homogeneous versus non-homogeneous; laminar versus non-laminar; separated versus non-separated; trabecular versus non-trabecular), (7) hematoma volume below versus above prespecified cut off values (121 mm^3^ [28]), and (8) hematoma MRI-hypo- and -iso-intensity versus hyper- and mixed intensity on T1, T2, and DW-imaging. Statistical heterogeneity in each meta-analysis was assessed using the T2, I2, and chi-square tests. When heterogeneity was moderate to high (*I*^2^ 50% or higher), a random effects model was used; if this was lower than 50%, a fixed effects model was applied.

## Results

**Fig. 2:**
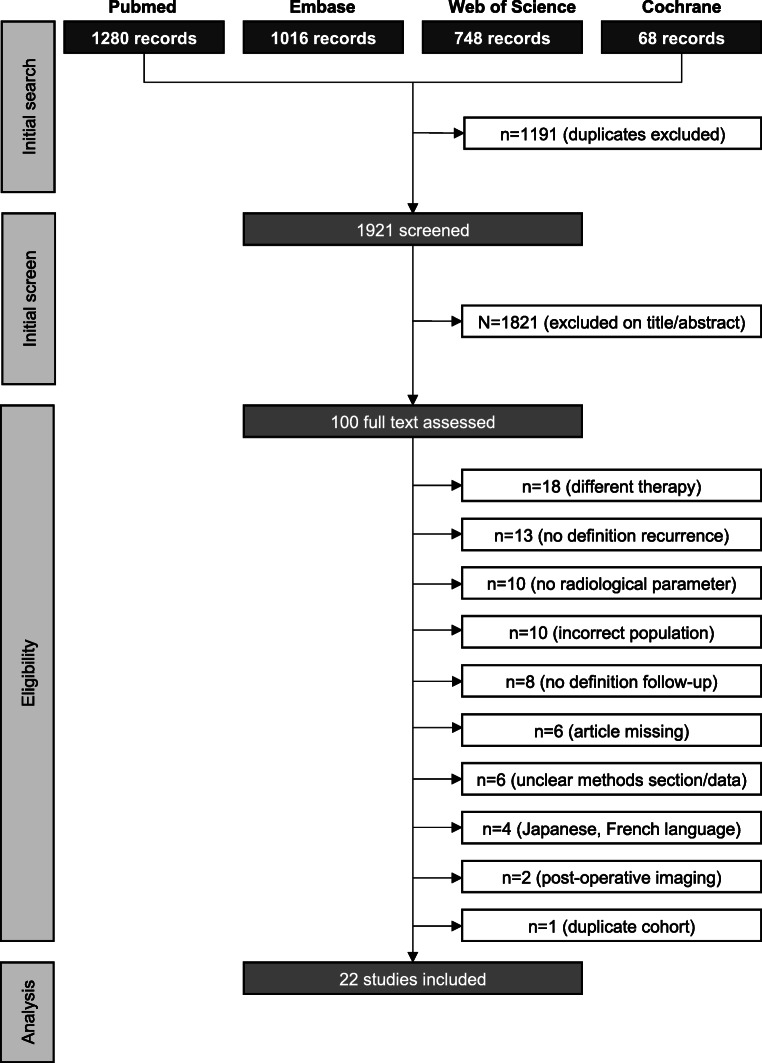
Flow-chart of included studies

We identified 3112 publications published between 1 January 1940 and September 2020, of which 100 were evaluated in full text and 22 finally included in the meta-analysis (Fig. 2 flow-chart of included studies). All studies scored three to four stars on the selection category of the NOS questionnaire. Scores on the outcome category varied between two to three, depending on the reporting on follow-up. None of the studies adjusted for confounders, resulting in no stars for the comparability section. Study quality was classified as fair for three (14%) and poor for 19 (86%) studies (Table 2).

**Table 2:** Newcastle–Ottawa Quality Assessment Scale (NOS), cohort studies

Study	Selection	Comparability	Outcome	Study quality
Won et al. 2020 [36]	★★★★	–	★★	Poor
Shen et al. 2019 [21]	★★★★	–	★★★	Fair
You et al. 2018 [27]	★★★★	–	★★	Poor
Yan et al. 2018 [28]	★★★	–	★★★	Poor
Lee et al. 2018 [44]	★★★★	–	★★	Poor
Bartek et al. 2017 [9]	★★★★	–	★★	Poor
Hammer et al. 2017 [43]	★★★	–	★	Poor
Kim et al. 2017 [45]	★★★★	–	★★	Poor
Han et al. 2017 [19]	★★★★	–	★★★	Fair
Stavrinou et al. 2017 [46]	★★★★	–	★★	Poor
Goto et al. 2015 [47]	★★★★	–	★★	Poor
Jung et al. 2015 [25]	★★★	–	★★	Poor
Song et al. 2014 [29]	★★★	–	★★	Poor
Jeong et al. 2014 [32]	★★★	–	★★	Poor
Huang et al. 2014 [30]	★★★★	–	★★★	Fair
Stanisic et al. 2013 [48]	★★★★	–	★★	Poor
Chon et al. 2012 [23]	★★★★	–	★★	Poor
Ko et al. 2008 [24]	★★★★	–	★★	Poor
Amirjamshidi et al. 2007 [20]	★★★	–	★★	Poor
Yamamoto et al. 2003 [49]	★★★★	–	★★	Poor
Nakaguchi et al. 2001 [26]	★★★	–	★★	Poor
Oishi et al. 2001 [50]	★★★★	–	★★	Poor

### Study and patient characteristics

All 22 studies were cohort studies, of which three (14%) had a prospective follow-up design (Table 3). Four definitions were identified for CSDH recurrence after primary surgery: (1) surgery (reoperation), without additional clinical or radiological criteria (*n* = 6); (2) clinical symptoms and/or radiological signs requiring additional surgery (*n* = 1); (3) combination of clinical recurrence or progression of symptoms and radiological recurrence or progression of ipsilateral CSDH (*n* = 10); and finally (4) only radiologic recurrence or progression of CSDH (*n* = 5). In three of these latter five studies, all patients received additional surgery due recurrent or progressive symptoms [29, 45, 49]. One study reported a reoperation in 16 out of 21 cases (76%) due to reappearance of symptoms with observation only in the remaining patients [26]. The fifth study mentioned a reoperation was performed if reappearance of symptoms accompanied the radiological CSDH recurrence, without describing the number of patients requiring surgery however [20]. An overview of the radiological parameters evaluated in this meta-analysis is provided in Table 3. Follow-up period ranged from 3 to 12 months. Six patients died prior to discharge [21, 45], leading to a total inclusion of 5566 CSDH patients in the meta-analysis with CSDH recurrence occurring in 801 (14.4%; Table 4). Overall male-female ratio was 3:1 with a mean age of 68.9 years (SD 4.1; *n* = 18 studies) and a precipitating head trauma in 2089 patients (62.6%; *n* = 17 studies). Fourteen hundred and thirty-eight patients had used anti-thrombotic agents (28.9%; *n* = 17 studies) with the use of anticoagulation in 517 (10.4%, *n* = 11 studies), platelet aggregation inhibitors (PAI) in 829 (18.1%, *n* = 10), and unspecified therapy in 92 patients (2.0%, *n* = 5 studies). All patients were treated by BHC with subdural drainage during 24 to 72 h (Table 4).

**Table 3:** Study characteristics

Study	Country	Period	Design	Patients (*n*)	Definition CSDH	Definition reCSDH	Follow-up (months)	Radiological parameter
Won et al. 2020 [36]	Germany	2016–2018	Retro.	389	No	S	3	L
Shen et al. 2019 [21]	China	2012–2018	Retro.	461^a^	No	C + R	3	L, T, M, D, A
You et al. 2018 [27]	China	2013–2016	Retro.	226	No	C + R + S	12	L, M, D, A
Yan et al. 2018 [28]	China	2010–2017	Retro.	231	No	C + R	3	L, T, M, D, A, V
Lee et al. 2018 [44]	Korea	2012–2015	Retro.	131	Yes^b^	C + R	6	D, MRI-DWI
Bartek et al. 2017 [9]	Sweden	2005–2010	Retro.	759	No	S	6	L, D
Hammer et al. 2017 [43]	Germany	2009–2012	Pros.	73	No	S	1.5	D, A
Kim et al. 2017 [45]	Korea	2010–2015	Retro.	248^c^	No	R	6	L, D
Han et al. 2017 [19]	Korea	2004–2014	Retro.	756	No	S	6	T, M
Stavrinou et al. 2017 [46]	Germany	2011–2014	Retro.	195	No	S	3	L, D, A
Goto et al. 2015 [47]	Japan	2004–2010	Retro.	414	No	C + R	6	L, MRI-T1 + T2
Jung et al. 2015 [25]	Korea	2008–2012	Retro.	182	No	S	12	L, M, D, A
Song et al. 2014 [29]	Korea	2009–2012	Retro.	97	Yes^b^	R	3	L, T, M, D
Jeong et al. 2014 [32]	Korea	2008–2012	Retro.	125	No	C + R	3	L, M, D
Huang et al. 2014 [30]	Taiwan	2005–2006	Retro.	94	Yes^d^	C + R	3	L, M
Stanisic et al. 2013 [48]	Norway	2008	Pros.	107	Yes^b^	C + R	7	L, M, D, A
Chon et al. 2012 [23]	Korea	2006–2011	Retro.	420	No	C + R	3	L, T, M, D, A
Ko et al. 2008 [24]	Korea	2001–2006	Retro.	255	Yes^b^	C + R	3	L, M, D
Amirjamshidi et al. 2007 [20]	Iran	2000–2006	Pros.	82	No	R	3	T, M, D
Yamamoto et al. 2003 [49]	Japan	1991–2000	Retro.	105	No	R	3	L, M
Nakaguchi et al. 2001 [26]	Japan	1989–1998	Retro.	106	Yes^b^	R	3	D, A
Oishi et al. 2001 [50]	Japan	1995–1999	Retro.	116	No	C + R	3	L, D

*A* architecture; *BHC* + *D* burr hole craniostomy combined with post-operative subdural closed drainage system, *C* clinical recurrence/progression of symptoms, *D* density, *L* laterality, *M* midline shift, *Pros* prospective, *R* radiologic recurrence/progression of CSDH, *Retro* retrospective, *S* surgery, *T* thickness

^a^Four patients died before discharge, therefor analyses were performed in 457 patients

^b^Definition CSDH: radiologic finding of subdural fluid collection with peri-operative confirmation of CSDH

^c^Two patients died before discharge, therefor analyses were performed in 246 patients

^d^Definition CSDH: Diagnosis is based on pre-defined radiologic criteria with peri-operative confirmation of CSDH

**Table 4:** Patient characteristics

Study	Patients (*n*)	Gender (M:F)	Age (year)	Trauma (*n*, %)	OAC (*n*, %)	PAI (*n*, %)	OAC or PAI (*n*, %)	reCSDH (*n*, %)
Won et al. 2020 [36]	389	250:139	–	202 (52)	183	–	–	104 (27)
Shen et al. 2019 [21]	461^a^	376:81	69	235 (51)	–	–	28 (6)	69 (15)
You et al. 2018 [27]	226	184:42	65	161 (71)	–	–	14 (6)	34 (15)
Yan et al. 2018 [28]	231	188:43	–	–	–	–	–	33 (14)
Lee et al. 2018 [44]	131	85:46	68	71 (54)	–	–	35 (27)	7 (5)
Bartek et al. 2017 [9]	759	514:245	74	–	116 (15)	194 (26)	310 (41)	85 (11)
Hammer et al. 2017 [43]	73	47:26	75	–	–	–	–	19 (26)
Kim et al. 2017 [45]	248^b^	173:73	69	187 (75)	6 (2)	53 (21)	59 (24)^c^	31 (13)
Han et al. 2017 [19]	756	574:182	68	–	81 (11)	220 (29)	301 (40)	104 (14)
Stavrinou et al. 2017 [46]	195	134:61	71	99 (51)	48 (25)	56 (29)	104 (53)	35 (18)
Goto et al. 2015 [47]	414	279:135	77	–	14 (3)	70 (17)	84 (20)	37 (9)
Jung et al. 2015 [25]	182	131:51	68	125 (69)	10 (5)	36 (20)	46 (25)	25 (14)
Song et al. 2014 [29]	97	64:33	70	61 (63)	–	–	–	16 (16)
Jeong et al. 2014 [32]	125	92:33	69	81 (65)	–	35 (28)	35 (28)	8 (6)
Huang et al. 2014 [30]	94	79:15	69	70 (74)	2 (2)	12 (13)	14 (15)	13 (14)
Stanisic et al. 2013 [48]	107	72:35	72	86 (80)	15 (14)	36 (34)	51 (48)	17 (16)
Chon et al. 2012 [23]	420	334:86	67	237 (56)	34 (8)	117 (28)	151 (36)	92 (22)
Ko et al. 2008 [24]	255	150:105	65	181 (71)	8 (3)	–	8 (3)	24 (9)
Amirjamshidi et al. 2007 [20]	82	67:15	59	52 (63)	–	–	–	10 (12)
Yamamoto et al. 2003 [49]	105	73:32	71	78 (74)	–	–	4 (4)	11 (10)
Nakaguchi et al. 2001 [26]	106	82:24	67	63 (28)	–	–	–	17 (16)
Oishi et al. 2001 [50]	116	84:32	72	100 (86)	–	–	11 (9)	10 (9)

*OAC* oral anticoagulation, *PAI* platelet aggregation inhibitor, *reCSDH* CSDH recurrence

^a^Four patients died before discharge, therefor analyses were performed in 457 patients

^b^Two patients died before discharge, therefor analyses were performed in 246 patients

^c^Because of rounding, percentages in combined “OAC or PAI” group may differ with one percent from the sum of “OAC” and “PAI”

### Imaging findings: hematoma laterality, thickness, and midline shift

**Fig. 3:**
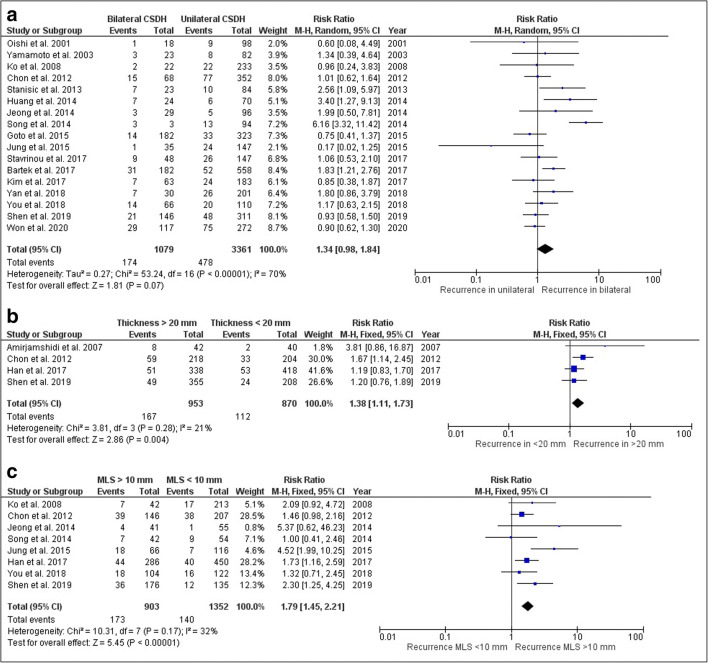
Forest plot on CSDH recurrence: **a** uni- versus bilateral hematoma; **b** hematoma thickness < or > 20 mm; **c** midline shift < or > 10 mm

Nineteen studies reported on uni- and bilaterality with incomplete data in two [43, 44], resulting in seventeen studies with a total of 4400 patients for laterality analysis with a high study heterogeneity (*I*^2^ = 70%). Patients with bilateral CSDH had higher hematoma recurrence than patients with a unilateral CSDH (Fig. 3a, RR 1.34, 95% CI 0.98–1.84).

Six studies with a total of 2150 patients reported on hematoma thickness using a cutoff value of 15 (*n* = 1), 20 (*n* = 4), or 25 (*n* = 1) mm. The largest group comparison showed that the recurrence rate of patients with a CSDH thickness of more than 20 mm was higher than patients with a hematoma thickness of less than 20 mm (Fig. 3b, RR 1.38, 95% CI 1.11–1.73). Adding the studies with cut off values of 15 or 25 mm, a similar result was seen (combined group: RR 1.46, 95% CI 1.19–1.79). Study heterogeneity was low in both comparisons (*I*^2^ = 21% and *I*^2^ = 5% respectively).

Thirteen studies with a total of 2874 patients described midline shift employing a cutoff value of 5 (*n* = 1), 10 (*n* = 8), or 15 mm (*n* = 1) or reported only on the presence or absence of midline shift (*n* = 3). Patients with a midline shift more than 10 mm had a higher recurrence rate than patients with a midline shift below 10 mm (Fig. 3c, RR 1.79, 95% CI 1.45–2.21). For the combined midline shift groups (adding results of 5 mm and 15 mm to 10 mm), recurrence risk remained significantly higher (RR 1.76, 95% CI 1.45–2.14). In the three studies describing only absence or presence of midline shift, there was no difference between the groups (RR 0.82, 95% CI 0.39–1.72). Study heterogeneity was low in all three comparisons (*I*^2^ = 32%, *I*^2^ = 14%, and *I*^2^ = 0 respectively).

### Imaging findings: hematoma density and architecture

**Fig. 4:**
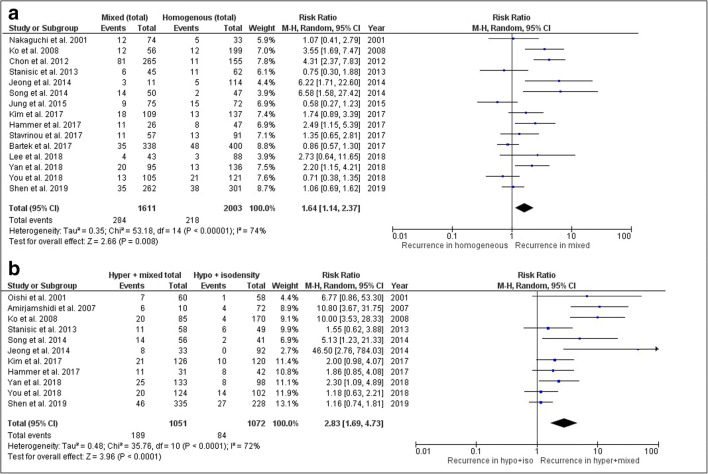
Forest plot on CSDH recurrence: **a** homogeneous versus mixed density hematoma; **b** iso- and hypodensity versus hyper- and mixed density hematoma

Seventeen studies with a total of 3813 patients reported on hematoma density. In fifteen studies (*n* = 3614), data were reported or could be reconstructed on homogeneity of the hematoma and mixed density categories. There was a higher risk of recurrence in patients with a mixed density hematoma than in patients with a (complete hypo-, iso-, or hyperdense) homogeneous hematoma (Fig. 4a, RR 1.64, 95% CI 1.14–2.37). Eleven studies reported on homogeneous iso- and hypodensity versus hyper- and total mixed density hematomas. Patients with hyper- and mixed density hematomas had more often CSDH recurrence than patients with homogeneous hypo- and isodensity hematomas (Fig. 4b, RR 2.38, 95% CI 1.69–4.73). Study heterogeneity was high in both comparisons (*I*^2^ = 74% and *I*^2^ = 71% respectively).

**Fig. 5:**
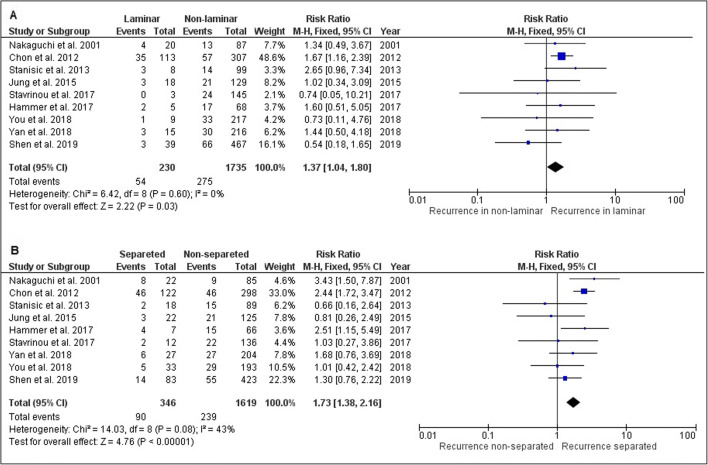
Forest plot on CSDH recurrence: **a** laminar hematoma architecture; **b** separated hematoma architecture

Nine studies with a total of 1965 CSDH patients reported on hematoma architecture by evaluation of all four predefined categories. Patients with laminar or separated architecture had a higher risk of hematoma recurrence than those with hematomas in which these features were not present (Fig. 5a, RR 1.37, 95% CI 1.04–1.80; and Fig. 5b, RR 1.76, CI 95% 1.38–2.16, respectively). Study heterogeneity was low in both comparisons (*I*^2^ = 0 and *I*^2^ = 43 respectively). There was no difference in hematoma recurrence for trabecular architecture (RR 0.88, 95% CI 0.52–1.49), with high study heterogeneity (*I*^2^ = 61%).

### Imaging findings: hematoma volume and MRI-sequences

One study (*n* = 514) reported on hematoma volume with frequencies above or below a prespecified cut off value of 121 mm^3^ based on the receiver operating characteristics (ROC) curve, with the highest recurrence rates in hematomas with a baseline volume above 121 mm^3^ [28].

Two studies described results on MRI-sequences in relation to CSDH recurrence. The first study (*n* = 414) reported data on the predictive value of MRI-T1 and -T2 sequences, revealing the T1 classification to be the only prognostic predictor for CSDH recurrence in T1-iso/hypo-intensity group relative to the T1-hyperintensity group [47]. The second study (*n* = 131) revealed more CSDH recurrence when baseline MRI showed DWI hyperintensity compared to hypo-intensity [44].

## Discussion

In this meta-analysis including over 5500 patients, we identified prognostic factors on CT for recurrence of surgically treated CSDH patients. Hyperdense and mixed density hematoma were associated with the highest risk of CSDH recurrence, as were laminar and separated architecture hematomas. In addition, CSDH with greater magnitude of hematoma thickness and midlines shift carried an increased risk for recurrence.

The establishment of radiological prognostic factors for CSDH recurrence is of importance in the identification of vulnerable symptomatic CSDH patients for poor outcome and retreatment [19, 20]. This population would benefit most from optimization of therapy. Many preoperative radiological parameters have been reported as prognostic factors for CSDH recurrence, but results are conflicting [21–34, 51]. Overall, we found homogeneous hyperdense and mixed density hematoma to be associated with increased recurrence rates. Hematoma density relative to brain parenchyma on a CT image represents the proportion of fresh blood, with hypodense areas representing hematoma of older age and hyperdense components of more recent or active bleeding [52–55]. This imaging appearance reflects the protein concentration from plasma exudation with higher concentration in hyperdense hematoma [26, 38, 50, 56, 57]. In experimental studies, blood evokes an inflammatory reaction in the subdural space [42, 58]. This inflammatory reaction is associated with a high amount of inflammatory markers and causes the CSDH to be more active and is presumed to play a part in hematoma persistence, a greater tendency for re-bleeding and recurrence [39, 58–61]. Novel experimental approaches have evaluated pharmacological adaption of endothelial barrier function, modifying endothelial permeability and plasma exudation [62, 63]. However, reproducible animal models of human CSDH are not established yet [42]. Results of this meta-analysis are in concordance with the abovementioned pathophysiology of protein concentration in the subdural space, which also explains why lower recurrence rates were found in iso- and hypodense CSDH than in hyperdense hematomas.

Besides categories that describe density of the hematoma, internal architecture types are also used for classification. An established and commonly used classification is that of Nakaguchi (homogeneous, laminar, separated, trabecular), corresponding to proposed stages in natural history of a CSDH [26]. Overall, we found a higher recurrence risk in laminar and separated hematoma than in other hematomas. Several individual studies, however, did not report a high recurrence rate in laminar hematoma [29, 43, 64], but did report trabecular hematoma, corresponding to hematoma with multiplicity of cavities, to reoccur more often [49, 65–69]. This variation and discrepancy is most likely caused by the many available architecture categories which are applied parallel to the classification of Nakaguchi (i.e., loculated hematoma, hematoma with multiplicity of cavities, layered type hematoma, organized hematoma, and niveau formation), but could also be due to difficulties in applying the classification correctly [18, 26, 45, 59, 70, 71]. In addition, complex internal architecture categories might be very informative, but application can lead to significant intra- and interobserver variability compromising generalizability. In this paper, we propose and demonstrate the benefit of a simplified hematoma classification system based on hematoma density solely. This comprises of a homogeneous iso- and hypodensity category and a second category of CSDH with hyperdense components. This simplified classification could be easy to apply in daily practice with presumably low inter- and intra-observer variation and good insight in the recurrence risk. Future research should confirm the significance of this finding, and also whether adding the different architecture subcategories yields valuable surplus information.

We demonstrate that a greater magnitude of hematoma thickness and a midline shift is associated with an increased recurrence risk. Increased CSDH size and midline shift are often attributed to brain atrophy in close relation to aging, providing the CSDH a potential space to increase easily [37, 40]. Previous studies have shown cerebral atrophy to be a potential risk factor for CSDH recurrence [20, 50]. The intracranial (counter-) pressure from brain volume reflects the elasticity of brain parenchyma and may play a part in optimal hematoma absorption [40, 41]. Due to a decrease in brain elasticity and counter pressure by advanced age and atrophy post-operative, re-expansion might potentially be less effective leaving a larger postoperative subdural space that could facilitate persistence or recurrence of CSDH [23, 41, 72]. This mechanism may also explain the increased recurrence in bilateral CSDH. In daily practice, grading of cerebral atrophy is a challenging and difficult task at the time of CSDH—diagnosis. The compression caused by the subdural hematoma on the involved hemisphere distorts the gyri sulci pattern due to the raised intracranial pressure and complicates a reliable assessment. Furthermore, several scales exist to classify atrophy, causing once again large inter- and intra-observer variation. Further evaluation of this parameter was therefore beyond the scope of this meta-analysis.

Recurrence risk is influenced by patient as well as radiological hematoma characteristics. Since a non-contrast CT scan is the most frequently performed diagnostic modality, evaluation of CT predictors is of great additional value next to other presumed clinical predictors such as age, concomitant chronic illness or coagulopathy [21, 23, 28, 64, 71, 73–75]. Similar to the limitations of studies evaluating the value of radiological predictors in recurrence risk, varying results have also been published regarding the effect of age, sex, anti-coagulant use, and chronic illness [33, 72, 76, 77]. The addition of radiological predictors of recurrences to baseline patient characteristics for risk calculation may facilitate clinicians to identify patients prone to recurrence more accurately. These findings could lead to adaptation of treatment measures on an individual basis in order to lower the recurrence risk, for example by postponing surgical drainage when hyperdense components are present or adjusting the (local standard) term for anticoagulant therapy resumption post-operative. Limited data also suggest that the addition of corticosteroids might be beneficial in reducing recurrence risk in high-risk patients [73].

Limitations of this meta-analysis are due to methodological aspects of the included studies. We encountered significant heterogeneity in the definitions used for CSDH recurrence, i.e., only radiological recurrence, or the combination of recurring symptoms with radiological persistence or progression of CSDH, or merely re-operation without clarifying the criteria for reoperation. Furthermore, differences in duration of follow-up, hematoma density, and architecture classification and measurement techniques for radiological parameters also contributed to data heterogeneity. Evaluation of study quality using the NOS questionnaire revealed that the majority of studies did not reach maximum quality scores, mainly because no adjustments were performed for confounding factors and incomplete follow up information. However, the findings were generally consistent and in line with acknowledged clinically relevant parameters.

For the present study, we included only surgically treated CSDH patients by burr hole or twist drill craniostomy with subdural drainage, the mainstay treatment worldwide, in order to eliminate the potential effect of different treatment strategies on recurrence rates. We maintained a study protocol with strict inclusion and exclusion criteria in order to achieve good quality and homogeneous data as good as possible, which to our knowledge has provided the first data review on this subject.

## Conclusion

From the present meta-analysis, we have derived several CT predictors that are associated with recurrence after surgical treatment of CSDH. In particular, CSDH with hyperdense components or with laminar or separated architecture type entail higher recurrence rates. Preoperative assessment of these parameters identifies a population with higher CSDH recurrence risk, and appreciation of these findings allows clinicians to apply an individualized management strategy. Future research is necessary to validate the prognostic value of these CT parameters in prospective studies and in particular investigate the value of a simplified density classification. Clear definitions and description of radiological measurement techniques are mandatory for a reliable evaluation.

## Data Availability

This systematic review and meta-analysis used already published data obtained from the literature search to conduct meta-analyses. No funding is received for this study.

## References

[1] Almenawer SA, Farrokhyar F, Hong C, Alhazzani W, Manoranjan B, Yarascavitch B, Arjmand P, Baronia B, Reddy K, Murty N, Singh S (2014). Chronic subdural hematoma management: a systematic review and meta-analysis of 34,829 patients. Ann Surg.

[2] Kudo H, Kuwamura K, Izawa I, Sawa H, Tamaki N (1992). Chronic subdural hematoma in elderly people: present status on Awaji Island and epidemiological prospect. Neurol Med Chir.

[3] Trotter W (1914). Chronic subdural hemorrhage of traumatic origin and its relation to pachymeningitis haemorhhagica interna. Br J Surg.

[4] Drapkin AJ (1991). Chronic subdural hematoma: pathophysiological basis for treatment. Br J Neurosurg.

[5] Bosche B, Molcanyi M, Noll T, Kochanek M, Kraus B, Rieger B, el Majdoub F, Dohmen C, Löhr M, Goldbrunner R, Brinker G (2013). Occurrence and recurrence of spontaneous chronic subdural haematoma is associated with factor XIII deficiency. Clin Neurol Neurosurg.

[6] Shim YS, Park CO, Hyun DK, Park HC, Yoon SH (2007). What are the causative factors for a slow, progressive enlargement of a chronic subdural hematoma. Yonsei Med J.

[7] Ito H, Komai T, Yamamoto S (1978). Fibrinolytic enzyme in the lining walls of chronic subdural hematoma. J Neurosurg.

[8] Labadie EL, Glover D (1975). Local alterations of hemostatic-fibrinolytic mechanisms in reforming subdural hematomas. Neurology.

[9] Bartek J, Sjåvik K, Kristiansson H, Stahl F, Fornebo I, Förander P (2017). Predictors of recurrence and complications after chronic subdural hematoma surgery: a population-based study. World Neurosurg.

[10] Liu W, Bakker NA, Groen RJ (2014). Chronic subdural hematoma: a systematic review and meta-analysis of surgical procedures. J Neurosurg.

[11] Sun TF, Boet R, Poon WS (2005). Non-surgical primary treatment of chronic subdural haematoma: preliminary results of using dexamethasone. Br J Neurosurg.

[12] Soleman JN, Mariani L (2017). The conservative and pharmacological management of chronic subdural haematoma. Swiss Med Wkly.

[13] Delgado-Lopez PD, Martin-Velasco V, Castilla-Diez JM, Rodriquez-Salazar A, Galacho-Harriero AM, Fernandex-Arconada O (2009). Dexamethasone treatment in chronic subdural haematoma. Neurocirugia.

[14] Miah IP, Herklots M, Roks G, Peul WC, Walchenbach R, Dammers R, Lingsma HF, den HM H, Jellema K, van der NA G (2019) Dexamethasone Therapy in Symptomatic Chronic Subdural Hematoma (DECSA-R): a retrospective evaluation of initial corticosteroid therapy versus primary surgery. J Neurotrauma 37:366–372 10.1089/neu.2019.6541 31452450

[15] Weigel R, Schmiedek P, Krauss JK (2003). Outcome of contemporary surgery for chronic subdural haematoma: evidence based review. J Neurol Neurosurg Psychiatry.

[16] Santarius T, Hutchinson PJ (2004). Chronic subdural haematoma: time to rationalize treatment?. Br J Neurosurg.

[17] Santarius T, Kirkpatrick PJ, Ganesan D, Chia HL, Jalloh I, Smielewski P, Richards HK, Marcus H, Parker RA, Price SJ, Kirollos RW, Pickard JD, Hutchinson PJ (2009). Use of drains versus no drains after burr-hole evacuation of chronic subdural haematoma: a randomised controlled trial. Lancet.

[18] Matsumoto HH, Okada T, Sakurai Y, Minami H, Masuda A, Tominaga S (2017). Clinical investigation of refractory chronic subdural hematoma: a comparison of clinical factors between single and repeated recurrences. World Neurosurg.

[19] Han MH, Ryu JI, Kim CH, Kim JM, Cheong JH, Yi HJ (2017) Predictive factors for recurrence and clinical outcomes in patients with chronic subdural hematoma. J Neurosurg 127:1117–1125 10.3171/2016.8.JNS16867 27982768

[20] Amirjamshidi AA, Eftekhar B, Rashidi A, Rezaii J, Esfandiari K, Shirani A (2007). Outcomes and recurrence rates in chronic subdural haematoma. Br J Neurosurg.

[21] Shen J, Yuan L, Ge R, Wang Q, Zhou W, Jiang XC, Shao X (2019). Clinical and radiological factors predicting recurrence of chronic subdural hematoma: a retrospective cohort study. Injury.

[22] Altaf IS, Vohra AH (2018). Radiolological predictors of recurrence of chronic subdural hematoma. Pak J Med Sci.

[23] Chon KH, Lee JM, Koh EJ, Choi HY (2012). Independent predictors for recurrence of chronic subdural hematoma. Acta Neurochir.

[24] Ko BSL, Seo BR, Moon SJ, Kim JH, Kim SH (2008). Clinical analysis of risk factors related to recurrent chronic subdural hematoma. J Korean Neurosurg Soc.

[25] Jung Y, Jung N, El K (2015). Independent predictors for recurrence of chronic subdural hematoma. J Korean Neurosurg Soc.

[26] Nakaguchi HT, Yoshimasu N (2001). Factors in the natural history of chronic subdural hematomas that influence their postoperative recurrence. J Neurosurg.

[27] You W, Zhu Y, Wang Y, Liu W, Wang H, Wen L, Yang X (2018). Prevalence of and risk factors for recurrence of chronic subdural hematoma. Acta Neurochir.

[28] Yan CY, Huang JW (2018). A reliable nomogram model to predict the recurrence of chronic subdural hematoma after burr hole surgery. World Neurosurgery.

[29] Song DHK, Chun HJ, Yi HJ, Bak KH, Ko Y, Oh SJ (2014). The predicting factors for recurrence of chronic subdural hematoma treated with burr hole and drainage. Korean J Neurotrauma.

[30] Huang YHL, Lu CH, Chen WF (2014). Volume of chronic subdural haematoma: is it one of the radiographic factors related to recurrence?. Injury.

[31] Huang YHY, Lee TC, Liao CC (2013). Bilateral chronic subdural hematoma: what is the clinical significance?. Int J Surg.

[32] Jeong SIK, Won YS, Kwon YJ, Choi CS (2014). Clinical analysis of risk factors for recurrence in patients with chronic subdural hematoma undergoing burr hole trephination. Korean J Neurotrauma.

[33] Tugcu B, Tanriverdi O, Baydin S, Hergunsel B, Gunaldi O, Ofluoglu E et al (2014) Can recurrence of chronic subdural hematoma be predicted? A retrospective analysis of 292 cases. J Neurol Surg A Cent Eur Neurosurg 75:37–41 10.1055/s-0032-1330961 23307307

[34] Jang KM, Chou HH, Mun HY, Nam TK, Park YS, Kwon JT (2020). Critical depressed brain volume influences the recurrence of chronic subdural hematoma after surgical evaluation. Nat Res Forum.

[35] Stang A (2010). Critical evaluation of the Newcastle-Ottawa scale for the assessment of the quality of nonrandomized studies in meta-analyses. Eur J Epidemiol.

[36] Won SY, Dubinski D, Eibach M, Gessler F, Herrmann E, Keil F et al (2020) External validation and modification of the Oslo grading system for predction of postoperative recurrence of chronic subdural hematoma. Neurosurg Rev:1–10. 10.1007/s10143-020-01271-w 10.1007/s10143-020-01271-w 32112162

[37] Spallone AG, Gagliardi FM, Vagnozzi R (1989). Chronic subdural hematoma in extremely aged patients. Eur Neurol.

[38] Tokmak MI, Bek S, Gokduman CA, Erdal M (2007). The role of exudation in chronic subdural hematomas. J Neurosurg.

[39] Weigel RH, Schilling L (2014). Vascular endothelial growth factor concentration in chronic subdural hematoma fluid is related to computed tomography appearance and exudation rate. J Neurotrauma.

[40] Fukuhara TG, Asari S, Ohmoto T, Akioka T (1996). The relationship between brain surface elastance and brain reexpansion after evacuation of chronic subdural hematoma. Surg Neurol.

[41] Sklar FH, Beyer CW, Clark WK (1980). Physiological features of the pressure-volume function of brain elasticity in man. J Neurosurg.

[42] D’Abbondanza JA, Macdonald RL (2014). Experimental models of chronic subdural hematoma. Neurol Res.

[43] Hammer AT, Kerry G, Schrey M, Hammer C, Steiner HH (2017). Predictors for recurrence of chronic subdural hematoma. Turk Neurosurg.

[44] Lee SHC, Lim DJ, Ha SK, Kim SD, Kim SH (2018). The potential of diffusion-weighted magnetic resonance imaging for predicting the outcomes of chronic subdural hematomas. J Korean Neurosurg Soc.

[45] Kim SUL, Kim YI, Yang SH, Sung JH, Cho CB (2017). Predictive factors for recurrence after burr-hole craniostomy of chronic subdural hematoma. J Korean Neurosurg Soc.

[46] Stavrinou P, Katsigiannis S, Lee JH, Hamisch C, Krischek B, Mpotsaris A, Timmer M, Goldbrunner R (2017). Risk factors for chronic subdural hematoma recurrence identified using quantitative computed tomography analysis of hematoma volume and density. World Neurosurg.

[47] Goto HI, Nomura M, Tanaka K, Nomura S, Maeda K (2015). Magnetic resonance imaging findings predict the recurrence of chronic subdural hematoma. Neurol Med Chir.

[48] Stanisic M, Hald J, Rasmussen IA, Pripp AH, Ivanovic J, Kolstad F (2013). Volume and densities of chronic subdural haematoma obtained from CT imaging as predictor of postoperative recurrence: a prospective study of 107 operated patients. Acta Neurochir.

[49] Yamamoto HH, Hamada H, Hayashi N, Origasa H, Endo S (2003). Independent predictors of recurrence of chronic subdural hematoma: results of multivariate analysis performed using a logistic regression model. J Neurosurg.

[50] Oishi MT, Tamatani S, Kitazawa T, Saito M (2001). Clinical factors of recurrent chronic subdural hematoma. Neurol Med Chir.

[51] Santarius TQ, Sivakumaran R, Kirkpatrick PJ, Kirollos RW, Hutchinson PJ (2010). The role of external drains and peritoneal conduits in the treatment of recurrent chronic subdural hematoma. World Neurosurg.

[52] Lee KSB, Bae HG, Doh JW, Yun IG (1997). The computed tomographic attenuation and the age of subdural hematomas. J Korean Med Sci.

[53] Sieswerda-Hoogendoorn T, Postema FAM, Verbaan D, Majoie CB, van RR R (2014). Age determination of subdural hematomas with CT and MRI: a systematic review. Eur J Radiol.

[54] Bergstrom ME, Levander B, Svendsen P (1977). Computed tomography of cranial subdural and epidural hematomas: variation of attenuation related to time and clinical events such as rebleeding. J Comput Assist Tomogr.

[55] Scotti GT, Melancon D, Belanger G (1977). Evaluation of the age of subdural hematomas by computerized tomography. J Neurosurg.

[56] Fujisawa HN, Tsuchida E, Ito H (1998). Serum protein exudation in chronic subdural haematomas: a mechanism for haematoma enlargement?. Acta Neurochir.

[57] Gorelick PB, Weisman SM (2005). Risk of hemorrhagic stroke with aspirin use: an update. Stroke.

[58] Frati AS, Mainiero F, Ippoliti F, Rocchi G, Raco A, Caroli E (2004). Inflammation markers and risk factors for recurrence in 35 patients with a posttraumatic chronic subdural hematoma: A prospective study. J Neurosurg.

[59] Nomura SK, Fujisawa H, Ito H, Nakamura K (1994). Characterization of local hyperfibrinolysis in chronic subdural hematomas by SDS-PAGE and immunoblot. J Neurosurg.

[60] Kitazono MY, Satoh H, Onda H, Matsumoto G, Fuse A, Teramoto A (2012). Measurement of inflammatory cytokines and thrombomodulin in chronic subdural hematoma. Neurol Med Chir.

[61] Nakamura ST (1989). Extraction of angiogenesis factor from chronic subdural haematomas. Significance in capsule formation and haematoma growth. Brain Inj.

[62] Bosche B, Molcanyl M, Rej S, Doeppner TR, Obermann M, Müller DJ (2016). Low-dose lithium stabilizes human endothelial barrier by decreasing MLC phosphorylation and universally augments cholinergic vasorelaxation capacity in a direct manner. Front Physiol.

[63] Bosche B, Schäffer M, Graf R, Härtel FV, Schäfer U, Noll T (2013). Lithium prevents early cytosolic calcium increase and secondary injurious calcium overload in glycolytically endothelial cells. Biochem Biophys Res Commun.

[64] Ohba SK, Nakagawa T, Murakami H (2013). The risk factors for recurrence of chronic subdural hematoma. Neurosurg Rev.

[65] Nayil KR, Sajad A, Zahoor S, Wani A, Nizami F, Laharwal M (2012). Subdural hematomas: an analysis of 1181 Kashmiri patients. World Neurosurg.

[66] Desai VRS, Britz GW (2017). Management of recurrent subdural hematomas. Neurosurg Clin N Am.

[67] Gelabert-Gonzalez MIP, Garcia-Allut A, Martinez-Rumbo R (2005). Chronic subdural haematoma: surgical treatment and outcome in 1000 cases. Clin Neurol Neurosurg.

[68] Tanikawa MM, Yamada K, Yamashita N, Matsumoto T, Banno T, Miyati T (2001). Surgical treatment of chronic subdural hematoma based on intrahematomal membrane structure on MRI. Acta Neurochir.

[69] El-Kadi HM, Kaufman HH (2000). Prognosis of chronic subdural hematomas. Neurosurg Clin N Am.

[70] Kang MSK, Kwon HJ, Cho SW, Kim SH, Youm JY (2007). Factors influencing recurrent chronic subdural hematoma after surgery. J Korean Neurosurg Soc.

[71] Mori KM (2001). Surgical treatment of chronic subdural hematoma in 500 consecutive cases: clinical characteristics, surgical outcome, complications, and recurrence rate. Neurol Med Chir.

[72] Torihashi K, Sadamasa N, Yoshida K, Narumi O, Chin M, Yamagata S (2008). Independent predictors for recurrence of chronic subdural hematoma: a review of 343 consecutive surgical cases. Neurosurgery.

[73] Qian ZY, Sun F, Sun Z (2017). Risk factors for recurrence of chronic subdural hematoma after burr hole surgery: potential protective role of dexamethasone. Br J Neurosurg.

[74] Motoie RK, Otsuji R, Ren N, Nagaoka S, Maeda K, Ikai Y (2018). Recurrence in 787 Patients with chronic subdural hematoma: retrospective cohort investigation of associated factors including direct oral anticoagulant use. World Neurosurg.

[75] Motiei-Langroudi RS, Shi S, Adeeb N, Gupta R, Griessenauer CJ, Papavassiliou E (2018). Factors predicting reoperation of chronic subdural hematoma following primary surgical evacuation. J Neurosurg.

[76] Stanisic MLJ, Mahesparan R (2005). Treatment of chronic subdural hematoma by burr-hole craniostomy in adults: influence of some factors on postoperative recurrence. Acta Neurochir.

[77] Adachi A, Higuchi Y, Fujikawa A, Machida T, Sueyoshi S, Harigaya K, Ono J, Saeki N (2014). Risk factors in chronic subdural hematoma: comparison of irrigation with artificial cerebrospinal fluid and normal saline in a cohort analysis. PLoS One.

